# Lifetime economic burden of hemophilia using a nationwide real-world healthcare data

**DOI:** 10.1371/journal.pone.0333683

**Published:** 2025-10-06

**Authors:** Joo-Young Byun, Jae-Hoon Jung, Suk-Chan Jang, Jamin Lim, Mihai Park, Eui-Kyung Lee

**Affiliations:** 1 Department of Surgery, Penn State College of Medicine, Hershey, Pennsylvania, United States of America; 2 School of Pharmacy, Sungkyunkwan University, Suwon, South Korea; Cleveland Clinic Lerner Research Institute, UNITED STATES OF AMERICA

## Abstract

**Background:**

Patients with hemophilia require lifelong treatment, but the real-world lifetime economic burden of hemophilia remains unclear. This study aims to estimate the lifetime economic burden of hemophilia using real-world data, accounting for cost variation by disease phase and over time.

**Methods:**

Male patients with hemophilia A (PwHa) or B (PwHb) recorded in South Korea’s Health Insurance Review and Assessment (HIRA) database from 2007 to 2022 were included. Survival was estimated using the rolling extrapolation method. A phase-specific costing approach was applied, distinguishing three phases: before hemophilic arthropathy (BH), after hemophilic arthropathy (AH), and 1 year before death (BD). Transition probabilities from BH to AH were calculated based on incidence rates of hemophilic arthropathy. Phase-specific annual costs were modeled using generalized estimating equations (GEEs), and predicted costs were multiplied by phase-specific probabilities from birth to estimated life expectancy to derive lifetime costs.

**Results:**

Estimated life expectancy for PwHa (n = 2,624) and PwHb (n = 664) in South Korea between 2007−2022 was 76.13 and 77.54 years, respectively. The incidence rate of hemophilic arthropathy was 0.090 cases/person-year [95% confidence interval, 0.084–0.097] for PwHa and 0.080 [0.070–0.092] for PwHb, yielding transition probabilities from BH to AH of 0.086 (PwHa) and 0.077 (PwHb). Mean annual costs were highest in the BD phase, followed by AH and BH phases: PwHa (BH: $11,331; AH: $27,271; BD: $27,985); PwHb (BH: $15,567; AH: $38,659; BD: $55,985). Compared to PwHa, PwHb incurred 1.37 and 1.42 times higher BH and AH costs, respectively (p < 0.05). Estimated lifetime cost was 1.76 times higher for PwHb ($22.25 million) than PwHa ($12.62 million) in hypothetical patients born in 2000.

**Conclusions:**

Hemophilia imposes a substantial economic burden across the lifespan, with PwHb incurring higher lifetime costs than PwHa. These real-world estimates can support more informed resource allocation and planning for hemophilia care.

## 1. Introduction

Hemophilia is a rare genetic bleeding disorder that primarily affects males and causes potentially life-threatening bleeding, along with frequent bleeding in the joints and muscles [1]. Recurrent joint bleeding results in permanent and irreversible joint damage, known as hemophilic arthropathy [2]. Hemophilic arthropathy causes long-term joint deterioration with physical consequences, such as chronic pain and disability, and reduces the quality of life of patients [3–5].

With early diagnosis and the onset of bleeding events occurring in childhood [1,4,6–8], treatment for hemophilia typically begins at a young age. Most treatment strategies focus on symptom relief or preventing disease progression through prophylaxis [1]. Due to the lack of curative options [9], patients with hemophilia require lifelong treatment [10]. Given that the life expectancy of patients with hemophilia in South Korea between 1991 and 2012 was reported to be approximately 69 years [11], the lifetime disease-related costs for these patients are expected to be substantial.

Several economic evaluation studies have estimated the lifetime costs of hemophilia using modeling approaches. However, these models begin at ages 12 [12,13], 18 [14,15], or even 34 years [16], thereby excluding costs incurred during early childhood. Given that hemophilia-related costs are substantial for patients aged ≤19 [17], these estimates may significantly underestimate the true lifetime economic burden. Furthermore, as treatment strategies vary widely depending on availability and disease severity, lifetime cost estimates based on a single treatment approach may not accurately reflect real-world practice. Previous models have also overlooked the impact of age-related disease progression and temporal trends in treatment costs over calendar years. Additionally, with growing recognition of potential clinical and outcome differences between hemophilia A and B [18], comparative analyses of disease progression and associated economic burden between the two types are warranted.

However, estimating lifetime costs using real-world data poses significant challenges due to heavy censoring and incomplete observations in both survival and cost data. Several methods have been proposed to address these limitations. Lin et al. [19] introduced a survival-adjusted estimator that partitions the follow-up period into small intervals; however, this approach may still introduce bias due to discretized censoring periods. Bang and Tsiatis [20] proposed an alternative method using inverse probability weighting, which accommodates continuous censoring without requiring interval partitioning. Yet, this approach effectively assigns zero costs beyond the censoring point, thereby relying only on uncensored data for cost estimation. To extrapolate survival using claims data, Hwang et al. [21,22] suggested a method based on applying a logit transformation of survival from a reference population to estimate survival in the target population. Furthermore, to address limitations associated with restricting the estimation period, Wijeysundera et al. [23] proposed a phase-based costing approach, which is particularly useful in the context of substantial censoring.

Given the lack of evidence on the real-world data-driven lifetime economic burden of hemophilia, developing robust lifetime cost estimates adjusted for censoring is essential for accurately quantifying disease burden and assessing the value of therapeutic innovations. This study aimed to estimate and compare the lifetime costs associated with hemophilia A and B, accounting for differences in life expectancy and treatment costs driven by disease progression (e.g., hemophilic arthropathy), aging, and calendar time in South Korea.

## 2. Materials and methods

### 2.1. Data source

This study used the 2007–2022 Health Insurance Review and Assessment (HIRA) database of South Korea. The authors first accessed the HIRA data for research purpose on October 10, 2023. The HIRA database provides demographic and clinical information linked to claims data, including diagnosis codes, procedure codes, medication usage, healthcare utilization, and costs for enrollees in the National Health Insurance Service of South Korea. Because South Korea has a unified national health insurance system, the HIRA database covers the entire nation of South Korea (approximately 51 million people). The HIRA database does not provide any information to the users that can be used for identifying individual participants during or after the data collection. This study was exempt from the Institutional Review Board of Sungkyunkwan University since it utilized anonymized retrospective data (SKKU202307042).

### 2.2. Population

Male patients diagnosed with and treated for hemophilia A (International Statistical Classification of Diseases and Related Health Problems 10^th^ Revision [ICD-10-CM]: D66) or B (ICD-10-CM: D67) between January 1, 2007 and November 30, 2022 were included in the study. Among them, patients who had a co-payment reimbursement code for hemophilia treatment (V009, V284) [24] during 2007–2022 were included and followed up from the first date of the reimbursement code (i.e., the first diagnosis of hemophilia). To show the overall age distribution of patients diagnosed with hemophilia in recent years, we presented the ages of patients with hemophilia in 2022.

### 2.3. Outcome measures and statistical analyses

#### 2.3.1. Survival estimation and life expectancy.

The rolling extrapolation method proposed by Hwang et al. [22] was used to estimate the survival function of patients with hemophilia beyond the observed period (from the first diagnosis of hemophilia to death or the end of data collection [November 30, 2022], whichever occurred first). According to the rolling extrapolation method, a method for extrapolating the survival function beyond the observed period in censored data, the relative survival function, W(t), between patients of interest (i.e., the target population) and the reference population (i.e., the general population) was estimated using the observed survival data of the target population and the survival data of the general population. Life expectancies were calculated using the width of the area under the estimated survival curves. Loss of life expectancy was defined as the difference between the width of the survival curve of the general male population and that of patients with hemophilia. Detailed explanation on rolling extrapolation is described in S1 File.

#### 2.3.2. Incidence rate of hemophilic arthropathy.

The incidence of hemophilic arthropathy was detected using the ICD-10-CM code (M362). The incidence rate was calculated as the time from the first diagnosis of hemophilia to the first incidence of hemophilic arthropathy. To capture newly diagnosed cases of hemophilic arthropathy, patients whose first diagnosis of hemophilic arthropathy occurred within 1 year of their initial hemophilia diagnosis were excluded from the incidence rate calculation. However, these patients were still included in the overall analysis population used for describing baseline characteristics, survival, and cost estimation. The incidence rate and its 95% confidence intervals (CIs) were calculated using a generalized linear model with Poisson distribution and log-link function. The incidence rate was then used to calculate the transition probability from ‘before hemophilic arthropathy’ to ‘after hemophilic arthropathy,’ with $p(t)=\text{1}-e^{-rt}$, where p is probability, r is incidence rate, and t is cycle length (1 year in our study) [25].

#### 2.3.3. Annual phase-specific cost estimation.

To reflect the differential clinical and economic burdens faced by patients by health status, a phase-specific cost estimation method has been used to estimate the lifetime economic burden [23]. Considering the substantially decreased quality of life and increased economic burden due to hemophilic arthropathy and death [26–28], we defined three phases (before hemophilic arthropathy, after hemophilic arthropathy, before death [from 1 year before death to death]) for hemophilia. Death was defined with diagnosis codes (e.g., R96: Other sudden death, cause unknown; R98: Unattended death; R99: Other ill-defined and unspecified causes of mortality; I461: sudden death) or in-hospital death codes. Additionally, patients who did not have any claims for more than five years during the observed period were defined as dead [29], assuming the last healthcare utilization date as the day of death. It is hard to eradicate hemophilic arthropathy once the symptom starts [2]; therefore, it was assumed that the transition from ‘after hemophilic arthropathy’ to ‘before hemophilic arthropathy’ does not happen. For each patient, longitudinal hemophilia-related cost data were categorized into three phases: before the onset of hemophilic arthropathy, after the onset of hemophilic arthropathy, and within one year prior to death. The cost data encompassed all hemophilia-related claims, including those related to both prophylactic and on-demand therapies. For robust cost estimation, we trimmed extreme cost data from the top and bottom 5% of the distribution in the analysis to reduce bias from outliers [30,31]. When estimating the mean or median phase-specific annual costs, the cost of the period with the possibility of misclassification was ruled out. For example, cost data from the early years (2007–2010) have the possibility of being misclassified into the ‘before hemophilic arthropathy’ phase with undetected previous diagnoses of hemophilic arthropathy before 2007. Moreover, cost data from late years (from 2022) could have been miscategorized into the ‘non-before death’ phase because death occurring after the study period could not be detected. The selected annualized phase-specific cost data were then used to fit generalized estimating equations (GEEs) to estimate phase-specific annual costs before and after the observed period. The November 2023 exchange rate was used to convert the Korean Won to the U.S. dollar (1292.2 Korean Won = 1 U.S. dollar).

#### 2.3.4. Lifetime cost estimation.

In our study, it was assumed that hemophilia treatment is initiated from birth and continues until death; therefore, treatment costs for hemophilia are incurred from birth to death. For each year, the annual treatment costs were estimated by multiplying the estimated probability of each phase by the corresponding phase-specific annual cost. Lifetime costs were calculated by summing the annual treatment costs from birth to the estimated life expectancy of patients with hemophilia. Hypothetical patients were assumed to have been born in 1990, 2000, and 2010, and were treated for hemophilia from birth to death. The lifetime cost for each hypothetical patient was estimated from the birth year to life expectancy.

#### 2.3.5. Patient characteristics.

To present the patients’ characteristics, the mean (standard deviation [SD]) and median (interquartile range [IQR]) were used as continuous variables, and the number of patients and percentages were used as categorical variables. The t-test and chi-squared test were used to assess significant differences between patients with hemophilia A and B.

For statistical analyses, we used SAS software, v.7.1 (SAS Institute, Cary, NC, U.S.A.).

## 3. Results

### 3.1. Patient characteristics

In total, 2,624 male patients with hemophilia A and 664 male patients with hemophilia B were included (Table 1). Patient characteristics were not significantly different between the hemophilia A and B groups. Over 70% of patients experienced incident hemophilic arthropathy during the observation period. The mean (SD) observation periods were 4,513 days (1,761 days) and 4,423 days (1,742 days) for hemophilia A and B, respectively. The proportion of patients who died during the observation period was 7.47% and 5.72% in the hemophilia A and B groups, respectively.

**Table 1:** Patient characteristics.

	Hemophilia A	Hemophilia B	p-value
**Total population 2007–2022, n**	2,624	664	
**Hemophilic Arthropathy, n (%)**^a^	1,932 (73.6)	469 (70.6)	0.120
**Observed period, days**^b^			0.236
Mean (SD)	4,513 (1,761)	4,423 (1,742)	
Median (IQR)	5,661 (3,310−5,801)	5,425 (3,097−5,794)	
**Death, n (%)**	196 (7.5)	38 (5.7)	0.118
**Prevalent patients in 2022, n**	2,435	628	
**Age of prevalent patients in 2022**			0.975
Mean (SD)	34 (18)	34 (19)	
Median (IQR)	32 (21-47)	32 (17-47)	

^a^ The numerator includes patients diagnosed with hemophilic arthropathy, while the denominator comprises all patients with hemophilia A or B. Note that the numerator reflects prevalent cases of hemophilic arthropathy, not newly diagnosed incident cases.

^b^ Observed period refers to time from the first diagnosis of hemophilia to death or end of the data (November 30, 2022), whichever occurred first.

Abbreviations: IQR, interquartile range; SD, standard deviation

In 2022, the last year of the study period, 2,435 and 628 patients had hemophilia A and B, respectively. The mean age (SD) of the patients in 2022 was 34.7 years (18.38 years) and 34.3 years (18.63 years) for the hemophilia A and B groups, with the median age being 32 years in both groups.

### 3.2. Incidence rate of hemophilic arthropathy

When assessing newly diagnosed hemophilic arthropathy, the incidence rate was 0.090 cases/person-year (95% CI, 0.084–0.097) for hemophilia A and 0.080 (95% CI, 0.070–0.092) for hemophilia B. Among those who with newly diagnosed incident hemophilic arthropathy (All: N = 1,012; hemophilia A: N = 789; hemophilia B: N = 223), the mean time from the first diagnosis of hemophilia and the first diagnosis of hemophilic arthropathy was significantly longer in the hemophilia B group than in the hemophilia A group (All: 1,563 days [SD 1,206 days]; hemophilia A: 1,466 days [SD 1,111 days]; hemophilia B: 1,903 days [SD 1,449 days], p < .001) (S1 Table, S1 Fig).

### 3.3. Life expectancy and phase distribution by time

**Fig 1:**
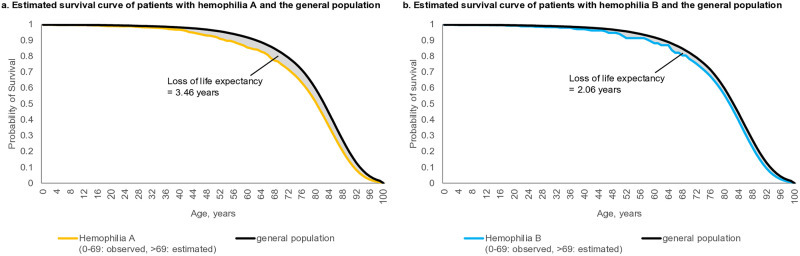
Life expectancy and loss of life expectancy in patients with hemophilia A and B compared to the general population.

The survival probability based on the observed and extrapolated survival data is shown in Fig 1. The estimated life expectancy was 76.13 years and 77.54 years for hemophilia A and B, respectively. Compared to the general population, which had an estimated life expectancy of 79.60 years, the loss of life expectancy was 3.46 and 2.06 years for hemophilia A and B, respectively.

**Fig 2:**
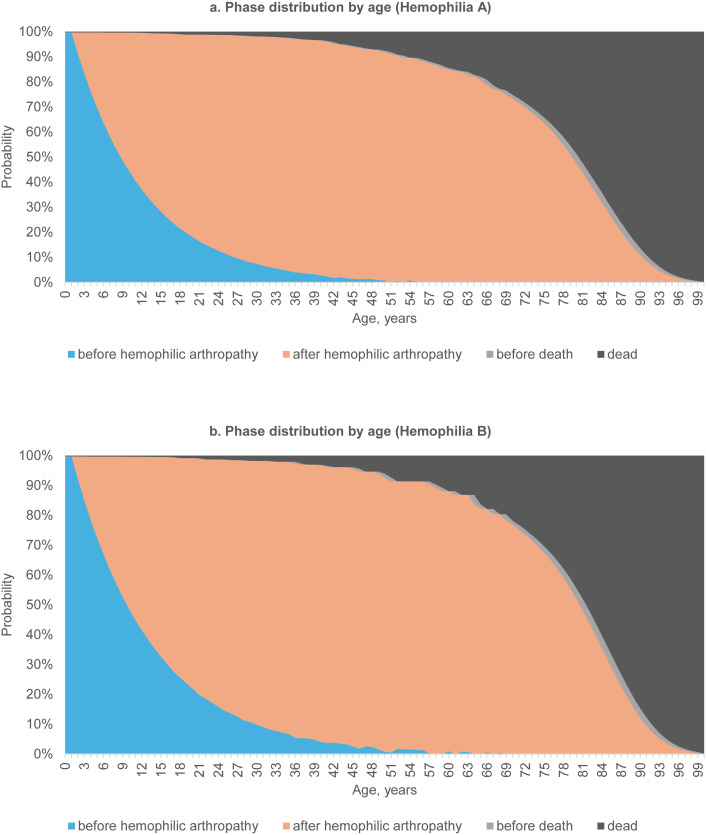
Phase distribution by age in patients with (a) hemophilia A and (b) hemophilia B.

Based on the estimated incidence rate of hemophilic arthropathy in section 3.2, the annual transition probability from the phase ‘before hemophilic arthropathy’ to ‘after hemophilic arthropathy’ was calculated to be 0.086 and 0.077 for hemophilia A and B, respectively. Fig 2 shows the phase distribution from age 0–100 based on the estimated survival probability and transition probability.

### 3.4. Phase-specific annual costs

The mean phase-specific annual cost for the ‘before hemophilic arthropathy, ‘after hemophilic arthropathy,’ and ‘before death’ phase was $11,331 (SD $14,783), $27,271 (SD $17,576), and $27,985 (SD $31,922) for hemophilia A and $15,567 (SD $20,934), $38,659 ($30,383), and $55,985 (SD $98,952) for hemophilia B, respectively (Table 2). The phase-specific annual cost of ‘before hemophilic arthropathy’ and ‘after hemophilic arthropathy’ was significantly higher in hemophilia B, showing 1.3 to 1.4 times higher costs compared to hemophilia A (p < 0.05). S2 Fig and S3 Fig show the phase-specific annual costs stratified by the year of cost occurrence and the lines of the GEE estimates.

**Table 2:** Phase-specific annual costs in Hemophilia A and B (U.S. dollars).

	Hemophilia A	Hemophilia B	p-value	Ratio
**Before hemophilic arthropathy**			0.001	
Mean (SD)	11,331 (14,738)	15,567 (20,934)		1.37
Median (IQR)	4,083 (540−18,102)	5,578 (376−23,238)		1.37
**After hemophilic arthropathy**			<.001	
Mean (SD)	27,271 (17,576)	38,659 (30,383)		1.42
Median (IQR)	25,872 (12,188−40,148)	34,720 (10,680−61,430)		1.34
**Before death**			0.166	
Mean (SD)	27,985 (31,922)	55,985 (98,952)		2.00
Median (IQR)	18,345 (5,524−36,679)	22,979 (10,540−43,197)		1.25

Ratio=cost of Hemophilia B/cost of Hemophilia A.

Abbreviations: IQR, interquartile range; SD, standard deviation.

### 3.5. Lifetime costs

Using estimates from GEEs, the estimated lifetime costs of hemophilia A were $7,068,429 for hypothetical patients born in 1990 and increased to $12,624,218 and $22,553,959 for hypothetical patients born in 2000 and 2010, respectively (Table 3). For hemophilia B, lifetime costs calculated with GEE estimates were $11,963,026, $22,250,382, and $41,410,833 for hypothetical patients born in 1990, 2000, and 2010, respectively. As a result, the lifetime costs were 1.69 to 1.84 times higher in hemophilia B than in hemophilia A. Considering that statistical significance for GEE models for ‘before death’ phase-specific annual costs was not achieved in both hemophilia A and B, lifetime costs estimated with GEE estimates for ‘before hemophilic arthropathy’ and ‘after hemophilic arthropathy’ phases and mean or median phase-specific annual cost for ‘before death’ phase are presented in S2 Table. The lifetime costs for an extended period (from birth to age 100 years) are presented in S3 Table.

**Table 3:** Lifetime costs of hemophilia A and B from birth to the estimated life expectancy using GEE estimates (U.S. dollars).

	Hemophilia A	Hemophilia B	Ratio
**Using GEE estimates (Patients born in 1990)**	7,068,429	11,963,026	1.69
**Using GEE estimates (Patients born in 2000)**	12,624,218	22,250,382	1.76
**Using GEE estimates (Patients born in 2010)**	22,553,959	41,410,833	1.84
**Using mean phase-specific annual costs**	1,738,191	2,491,295	1.43
**Using median phase-specific annual costs**	1,563,078	2,111,245	1.35

Ratio=cost of Hemophilia B/cost of Hemophilia A.

Abbreviations: GEE, generalized estimating equation.

## 4. Discussion

In the scarce evidence on the real-world lifetime economic burden in patients with hemophilia, our study shows that the lifetime cost of hemophilia is estimated to be $12.62 million and $22.25 million for patients with hemophilia A and B born in 2000, respectively. Considering the average lifetime healthcare cost per capita in South Korea, which was $92,340 in 2012 [32], the lifetime economic burden posed by hemophilia is substantial, with approximately 136 and 240 times the average lifetime health expenditure per capita in South Korea for hemophilia B and A, respectively.

In a previous study conducted in South Korea, the life expectancy was reported to be 69.03 years and 69.51 years in hemophilia A and B, respectively, resulting in an 8-year loss of life expectancy compared to the general male population in 2010 [11]. Their study used data from patients with hemophilia A and B from 1991 to 2012 without mentioning the exact data source. Compared to the previous study, our research reports a relatively longer life expectancy (76.13 years, 77.54 years for hemophilia A and B) and a lower loss of life expectancy (3.46 for hemophilia A and 2.06 years for hemophilia B). This improvement in life expectancy in our study could be attributed to the use of more recent data from 2007 to 2022. A similar trend of improved median life expectancy in patients with hemophilia over time was also observed in a Dutch study, in which life expectancy increased from 66 years (1973–1986) to 77 years (2001–2018) [33]. The development of more effective treatment strategies may contribute to improved survival of patients with hemophilia over time. For example, between 2014 and 2017, new drugs, such as efmororctocog alfa, rurioctocog alfa, lonoctocog alfa, eftrenonacog alfa, and emicizumab, were used for hemophilia treatment.

Although prophylaxis can delay the onset of hemophilic arthropathy, approximately 20% of young patients with severe hemophilia still develop symptoms despite prophylactic treatment [34]. Without appropriate management, hemophilic arthropathy commonly develops by the age of 20 in patients with severe hemophilia [35]. Even with intensive prophylaxis, joint bleeding persists in many young patients, suggesting that most individuals with hemophilia will experience some degree of hemophilic arthropathy by their 30s or 40s [36]. Similarly, in our study, the age-specific phase distribution showed that the majority of surviving patients had developed hemophilic arthropathy after age 50. Considering that our study population included patients with mild, moderate, and severe hemophilia, it can be inferred that, on average, hemophilic arthroaphty becomes prevalent around the age of 50 across the hemophilia spectrum.

Hemophilic arthropathy requires a range of interventions, including physiotherapy, activity pacing, exercise programs, assistive devices [37], pain management, immediate factor replacement, joint replacement surgery, and synovectomy [4,38]. As a result, the onset of hemophilic arthropathy is associated with significantly higher healthcare costs [17,27]. In South Korea, the rate of joint procedure, used to manage severe joint bleeding, is estimated at 0.02 to 0.06 per patient-year among patients with hemophilia A [39]. Based on this rate, it can be inferred that each year, among 1,000 patients with hemophilia, approximately 80–90 develop new cases of hemophilic arthropathy, and 20–60 undergo joint procedures. Consistently, our study found that the mean annual cost in the “after hemophilic arthropathy” phase was 2.4 to 2.5 times higher than in the “before hemophilic arthropathy” phase, indicating a substantial increase in economic burden following the onset of hemophilic arthropathy. By defining disease phases based on hemophilic arthropathy and incorporating real-world incidence rates, our analysis more accurately captures the differential healthcare expenditures across disease progression and improves the estimation of lifetime economic burden in hemophilia.

Previous economic evaluation studies have reported a wide range of lifetime costs for hemophilia, depending on the patient characteristics, treatment strategies, and analytical perspectives. From a U.S. healthcare perspective, lifetime costs for patients with hemophilia A with inhibitors (aged <12years) were estimated at $20.7 million (emicizumab prophylaxis), $31.0 million (no prophylaxis), and $99.2 million (bypassing agent prophylaxis) [12]. For patients with severe hemophilia A entering the model at age 34, lifetime costs were estimated at $11.2 million (factor VIII prophylaxis) and $22.1 million (efanescotocog alfa prophylaxis) [16]. In Canada, estimated lifetime costs for patients with severe hemophilia A (mean age 38) were CAD $33.2 million (~ USD $24.4 million) for emicizumab, CAD $34.7 million (~ USD $25.5 million) for extended half-life factor VIII, CAD $35.0 million (~ USD $25.7 million) for standard half-life factor VIII, and CAD $67.8 million (~ USD $49.8 million) for efanesocotog alfa [40].

For moderate or severe hemophilia B without inhibitors, lifetime costs were estimated at $12.1 million (etracogene dezaparvovec) and $23.2 million (factor IX) for adult patients in the U.S. [14], and at €5.3 million (~ USD $6.2 million; rFIX Fc fusion protein) and €6.3 million (~ USD $7.3 million; rFIX on-demand) for individuals aged ≥12 in Italy [13]. Similarly, in Germany, lifetime costs were estimated at €5.2 million (~ USD $6.1 million; etracogene dezaparvovec) and €6.4 million (~ USD $7.6 million; extended half-life factor IX) for adult patients with moderate to severe hemophilia B [15].

Given that pharmaceutical prices in South Korea are approximately 25.6%, 58.5%, 75.2% and 68.5% of those in the U.S., Canada, Germany, and Italy, respectively [41], direct comparison of absolute lifetime costs across countries may be inappropriate. Nonetheless, after accounting for international price differences, the lifetime economic burden estimated in our study appears generally higher than that reported in prior studies. This discrepancy may partly be explained by our incorporation of temporal trends in treatment costs using GEEs. While most previous models applied a constant discount rate to future costs, capturing time preferences, they did not account for dynamic cost changes over time due to therapeutic advancements or policy shifts. In contrast, our study captured observed trends of both increasing and decreasing phase-specific annual costs across calendar years. Applying these year-specific cost estimates resulted in lifetime costs that were approximately 6–8 times higher than those estimated using static mean and median annual costs throughout the lifetime. This finding suggests that ignoring cost changes over time may lead to substantial underestimation of economic burden over long time horizons.

Differences in study design, such as the characteristics of the study population (e.g., hemophilia severity, presence of inhibitors, treatment type), starting age (e.g., 12, 18, or 34 years in prior models), and analytical perspective, also likely contributed to cost discrepancies. Because previous studies estimated lifetime costs from the point of initiating a specific treatment strategy or after a certain age, their estimates do not fully capture the costs incurred during early childhood or the heterogeneity of real-world clinical management. In contrast, our study estimated lifetime costs from birth and accounted for variability in disease severity and treatment strategies, providing a more comprehensive view of the real-world economic burden of hemophilia across entire lifespan.

Studies comparing the costs of hemophilia A and B have reported mixed findings, varying by country and healthcare settings. In China and Portugal, higher costs were observed for hemophilia B, 2.4 and 1.4 times higher than for hemophilia A, respectively [42,43]. In the Chinese study, generalized linear model analyses indicated that patients with hemophilia A incurred 41.7% lower costs than those with hemophilia B. In contrast, studies from Taiwan and the U.S. reported higher treatment costs for hemophilia A, with costs 1.4 to 1.7 times greater than for hemophilia B [44,45].

Considering coagulation factor products account for approximately 90% of the total treatment costs for hemophilia [3,46,47] and similar units of coagulation factor are typically used for both hemophilia A and B [48], the unit price of coagulation factors in each healthcare system likely contributes to these cost difference. In South Korea, the price of recombinant coagulation factor for hemophilia B is approximately 1.4 times higher than that for hemophilia A [49,50]. Prior research from South Korea also reported that the per-patient cost of coagulation factor was about 1.5 times higher in hemophilia B than in hemophilia A [48]. These findings align with our study, where phase-specific costs for hemophilia B were approximately 1.4 times higher than those for hemophilia A, suggesting that the higher unit price of coagulation factors for hemophilia B may be a key driver of this cost difference in the South Korean context. In addition to pricing, factors such as extended hospital stays or suboptimal use of coagulation products may also contribute to the elevated costs observed for hemophilia B [42].

Our study has several limitations. First, as the analysis was conducted from the perspective of the South Korean National Health Insurance Service, the cost estimates may not be directly generalizable to other countries or healthcare systems. Nevertheless, our findings on cost comparisons between hemophilia types, disease phases, and temporal cost trends may still provide valuable insights for international researchers. Second, due to the lack of clinical data on hemophilia severity (e.g., blood clotting factor levels) in the claims data, we were unable to stratify patients by disease severity. Our hypothetical cohort includes all diagnosed hemophilia cases, so the estimated incidence of hemophilic arthropathy and lifetime costs represent averages across severity levels. Given that the prevalence of hemophilic arthropathy varies by hemophilia severity [51,52], and that patients with asymptomatic or undiagnosed mild hemophilia are likely underrepresented in claims data, the true incidence rate of hemophilic arthropathy in the general hemophilia population may be lower than our estimates suggest. Thus, our findings primarily reflect patients with clinically significant symptoms requiring intervention. Future studies using datasets with more detailed clinical information could provide better stratification of disease severity and more precise estimates of economic burden by severity level. Third, to project phase-specific annual costs beyond the observed period, we applied GEE models based on observed cost trends. As a result, our estimates may not capture unforeseen cost fluctuations due to unpredictable events such as pandemics or the introduction of novel therapies [53]. While we assumed that historical cost trends would continue, there remains a possibility of over- or underestimation, as future cost trajectories may deviate from past patterns. Fourth, we assumed a constant incidence rate of hemophilic arthropathy across age groups, due to the lack of age-specific incidence data. Fifth, we did not distinguish newly diagnosed hemophilia cases because of small sample sizes. However, by excluding patients with preexisting hemophilic arthropathy from the incidence rate calculation, we may have improved the accuracy of our incidence estimates. Lastly, due to limited mortality information, we defined death using a combination of diagnosis codes, diagnostic outcome codes, and prolonged absence of healthcare utilization. Despite this indirect approach, the life expectancy estimated in our study is consistent with recent reports [33], suggesting a low risk of significant misclassificaiton.

## 5. Conclusions

Based on trends in annual treatment costs observed in South Korean real-world data, the estimated lifetime costs per person exceed $12 million for hemophilia A and $22 million for hemophilia B. These estimates amount to roughly 136 and 240 times the national average for lifetime healthcare spending per capita in South Korea, with hemophilia B imposing a particularly higher financial burden. The significant lifetime financial burden highlighted in our study underscores the need for sufficient funding and resource allocation to support the long-term care and treatment of individuals with hemophilia.

## Supporting information

S1 File Method. Rolling extrapolation. (DOCX)

S1 Table Time to hemophilic arthropathy. (DOCX)

S2 Table Lifetime costs of hemophilia A and B from birth to estimated life expectancy (U.S. dollars). (DOCX)

S3 Table Lifetime costs of hemophilia A and B from birth to age 100 (U.S. dollars). (DOCX)

S1 Fig Kaplan-Meier curve of time to hemophilic arthropathy. (DOCX)

S2 Fig Distribution of phase-specific annual cost of hemophilia A by year and fitted generalized estimating equations (From left to right: before hemophilic arthropathy, after hemophilic arthropathy, and before death). (DOCX)

S3 Fig Distribution of the phase-specific annual cost of hemophilia B by year and fitted generalized estimating equations (From left to right: before hemophilic arthropathy, after hemophilic arthropathy, and before death). (DOCX)

S1 Data Supporting information. (DOCX)
