# Supplementary material for: Lifetime economic burden of hemophilia using a nationwide real-world healthcare data
Source: PLoS One. 2025 Oct 6;20(10):e0333683. doi: 10.1371/journal.pone.0333683 (PMC12500110; doi:10.1371/journal.pone.0333683)
Supplement: S1 File — (DOCX) [file pone.0333683.s001.docx]

**S1 Method. Rolling extrapolation**

Relative survival data during the observation period were used to fit the logit-transformed relative survival function, logit W(t). Considering that the logit W(t) tends to decline drastically in the initial period and progressively levels out to an almost straight line, the restricted cubic spline model is considered sufficiently flexible to fit logit W(t). The logit W(t) beyond the observed period was extrapolated with the fitted cubic spline model [1]. Following the rolling extrapolation method, we first estimated the survival function of the general male population by age based on the Kaplan-Meier method using the 2022 life table of the South Korean male population provided by Statistics Korea [2]. Considering that the life expectancy of Korean patients with hemophilia is 69 years [3], it was assumed that using the observed survival data from ages 0 to 69 years for the analysis could represent the survival of the Korean hemophilia population. Therefore, the survival function of patients with hemophilia aged 0–69 years was fitted using the survival data of patients with hemophilia from the HIRA database. For age points with a higher survival probability than the general male population, the survival probability of patients with hemophilia was substituted for that of the general male population. We then calculated the ratio between the survival curve of the general male population and that of patients with hemophilia, and the logit-transformed ratios at each age point were used to fit a restricted cubic spline model. The fitted restricted cubic spline model was used to predict the survival probability of patients aged 70 years with hemophilia. For the next step, a restricted cubic spline model was fitted with logit-transformed ratios of age 1-70 to predict the survival probability at the age of 71. The procedure was repeated until the age of 100 years, at which time the survival probability of the general male population was assumed to be 0.

**Reference**

1. Hwang, J.S., et al., *Estimating lifetime medical costs from censored claims data.* Health Econ, 2017. **26**(12): p. e332-e344.

2. Korea, S. *life table*. 2022 [cited 2024 April 15]; Available from: <https://kostat.go.kr/board.es?mid=a10301060900&bid=208&act=view&list_no=428312&tag=&nPage=1&ref_bid>=.

3. Yoo, K.Y., et al., *Life expectancy of Korean haemophiliacs, 1991-2012.* Haemophilia, 2014. **20**(4): p. e356-8.
