# Supplementary material for: Lifetime economic burden of hemophilia using a nationwide real-world healthcare data
Source: PLoS One. 2025 Oct 6;20(10):e0333683. doi: 10.1371/journal.pone.0333683 (PMC12500110; doi:10.1371/journal.pone.0333683)
Supplement: S1 Table — (DOCX) [file pone.0333683.s002.docx]

**S1 Table. Time to hemophilic arthropathy**

|  | **All** | **Hemophilia A** | **Hemophilia B** | **P-value** |
| --- | --- | --- | --- | --- |
| **Patients with incident hemophilic arthropathy, n** | 1,012 | 789 | 223 |  |
| **Observed period, days^a^** |  |  |  | 0.847 |
| Mean (SD) | 5,177 (1,180) | 5,181 (1,185) | 5,164 (1,165) |  |
| Median (IQR) | 5,775 (5,221-5,802) | 5,780 (5,250-5,803) | 5,760 (5,175-5,800) |  |
| **Time to hemophilic arthropathy, days^a^** |  |  |  | <.001 |
| Mean (SD) | 1,563 (1,206) | 1,466 (1,111) | 1,903 (1,449) |  |
| Median (IQR) | 1,104 (679-2,020) | 1,068 (647-1,930) | 1,460 (744-2,703) |  |

a: Calculated only with patients with incident hemophilic arthropathy.

Abbreviations: IQR, interquartile range; SD, standard deviation
