# Supplementary material for: Lifetime economic burden of hemophilia using a nationwide real-world healthcare data
Source: PLoS One. 2025 Oct 6;20(10):e0333683. doi: 10.1371/journal.pone.0333683 (PMC12500110; doi:10.1371/journal.pone.0333683)
Supplement: S2 Table — (DOCX) [file pone.0333683.s003.docx]

**S2 Table.** **Lifetime costs of hemophilia A and B from birth to estimated life expectancy (U.S. dollars)**

|  | **Hemophilia A** | **Hemophilia B** | **Ratio** |
| --- | --- | --- | --- |
| **Using GEE estimates for BH, AH, and mean phase-specific annual cost for BD (Patients born in 1990)** | 6,931,185 | 11,980,479 | 1.73 |
| **Using GEE estimates for BH, AH, and mean phase-specific annual cost for BD (Patients born in 2000)** | 12,347,524 | 22,269,915 | 1.80 |
| **Using GEE estimates for BH, AH, and mean phase-specific annual cost for BD (Patients born in 2010)** | 22,006,538 | 41,431,306 | 1.88 |
| **Using GEE estimates for BH, AH, and median phase-specific annual cost for BD (Patients born in 1990)** | 6,927,435 | 11,967,951 | 1.73 |
| **Using GEE estimates for BH, AH, and median phase-specific annual cost for BD (Patients born in 2000)** | 12,343,773 | 22,257,388 | 1.80 |
| **Using GEE estimates for BH, AH, and median phase-specific annual cost for BD (Patients born in 2010)** | 22,002,788 | 41,418,779 | 1.88 |

Ratio=cost of Hemophilia B/cost of Hemophilia A

Abbreviations: AH, after hemophilic arthropathy; BD, before death; BH, before hemophilic arthropathy; GEE, generalized estimating equation
