# Supplementary material for: Lifetime economic burden of hemophilia using a nationwide real-world healthcare data
Source: PLoS One. 2025 Oct 6;20(10):e0333683. doi: 10.1371/journal.pone.0333683 (PMC12500110; doi:10.1371/journal.pone.0333683)
Supplement: S3 Table — (DOCX) [file pone.0333683.s004.docx]

**S3 Table. Lifetime costs of hemophilia A and B from birth to age 100 (U.S. dollars)**

|  | **Hemophilia A** | **Hemophilia B** | **Ratio  (Hemophilia B /Hemophilia A)** |
| --- | --- | --- | --- |
| **Using GEE estimates (Patients born in 1990)** | 11,692,048 | 18,645,783 | 1.59 |
| **Using GEE estimates (Patients born in 2000)** | 21,001,903 | 34,653,071 | 1.65 |
| **Using GEE estimates (Patients born in 2010)** | 37,752,652 | 64,429,843 | 1.71 |
| **Using mean phase-specific annual costs** | 1,892,654 | 2,682,772 | 1.42 |
| **Using median phase-specific annual costs** | 1,704,624 | 2,328,923 | 1.37 |

Ratio=cost of Hemophilia B/cost of Hemophilia A

Abbreviations: GEE, generalized estimating equation
