# Supplementary figures and images for: Lifetime economic burden of hemophilia using a nationwide real-world healthcare data
Source: PLoS One. 2025 Oct 6;20(10):e0333683. doi: 10.1371/journal.pone.0333683 (PMC12500110; doi:10.1371/journal.pone.0333683)

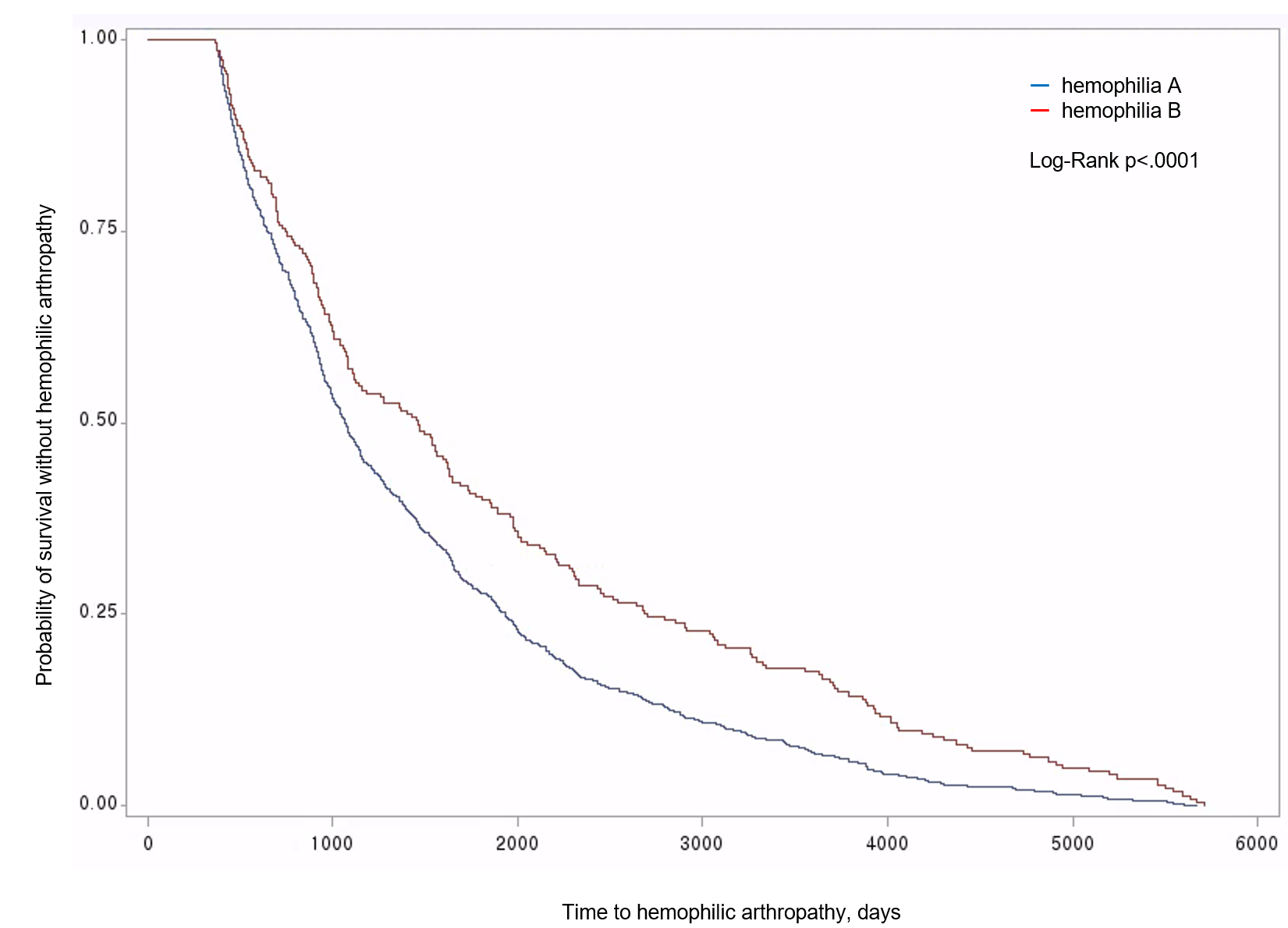


**S1 Fig. Kaplan-Meier curve of time to hemophilic arthropathy**

Supplement: S1 Fig — (DOCX) [file pone.0333683.s005.docx]
