# Supplementary material for: Lifetime economic burden of hemophilia using a nationwide real-world healthcare data
Source: PLoS One. 2025 Oct 6;20(10):e0333683. doi: 10.1371/journal.pone.0333683 (PMC12500110; doi:10.1371/journal.pone.0333683)
Supplement: S3 Fig — (DOCX) [file pone.0333683.s007.docx]

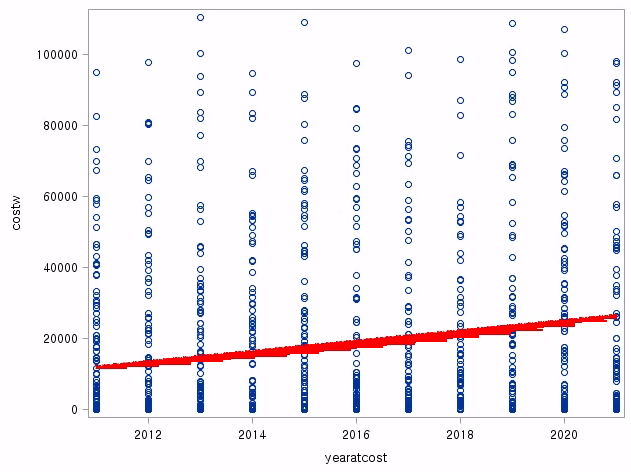

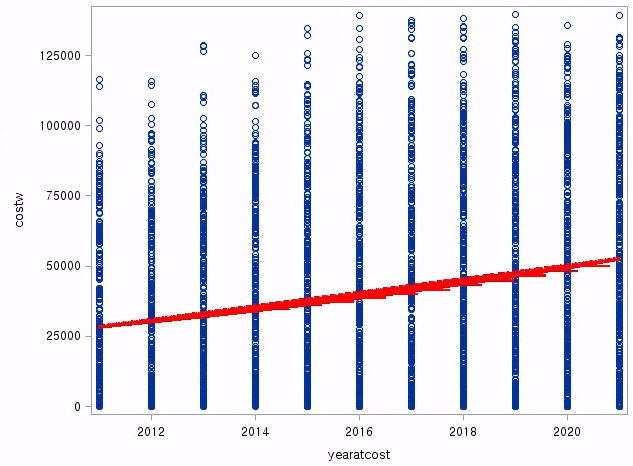

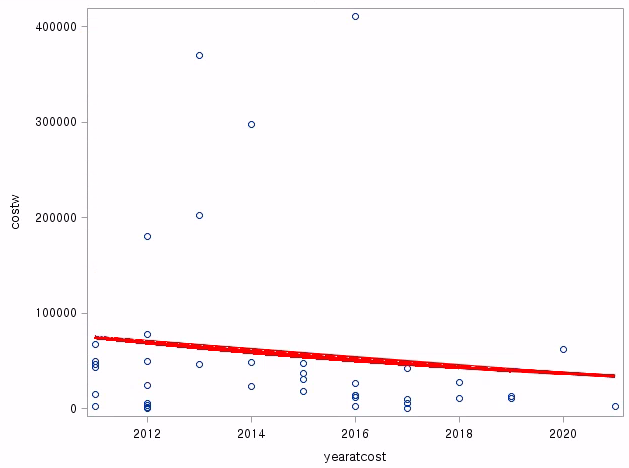


**S3 Fig.** Distribution of the phase-specific annual cost of hemophilia B by year and fitted generalized estimating equations (From left to right: before hemophilic arthropathy, after hemophilic arthropathy, and before death)
